# Near Infrared-Activated Dye-Linked ZnO Nanoparticles Release Reactive Oxygen Species for Potential Use in Photodynamic Therapy

**DOI:** 10.3390/ma13010017

**Published:** 2019-12-18

**Authors:** Jaspreet Singh Nagi, Kenneth Skorenko, William Bernier, Wayne E. Jones, Amber L. Doiron

**Affiliations:** 1 Department of Electrical and Biomedical Engineering, University of Vermont, Burlington, VT 05405, USA; Jaspreet-Singh.Nagi@uvm.edu; 2 ChromaNanoTech LLC, Binghamton, NY 13902, USA; kenneth@chromananotech.com (K.S.); wbernier@chromananotech.com (W.B.); 3 Department of Chemistry, Binghamton University (SUNY), Binghamton, NY 13902, USA; wayne.jones@unh.edu; 4 Provost and Vice President for Academic Affairs, University of New Hampshire, Durham, NH 03824, USA

**Keywords:** cytotoxicity, poly (ethylene glycol), cell viability, surface modification, photosensitizer

## Abstract

Novel dye-linked zinc oxide nanoparticles (NPs) hold potential as photosensitizers for biomedical applications due to their excellent thermal- and photo-stability. The particles produced reactive oxygen species (ROS) upon irradiation with 850 nm near infrared (NIR) light in a concentration- and time-dependent manner. Upon irradiation, ROS detected in vitro in human umbilical vein endothelial cells (HUVEC) and human carcinoma MCF7 cells positively correlated with particle concentration and interestingly, ROS detected in MCF7 was higher than in HUVEC. Preferential cytotoxicity was also exhibited by the NPs as cell killing was higher in MCF7 than in HUVEC. In the absence of irradiation, dye-linked ZnO particles minimally affected the viability of cell (HUVEC) at low concentrations (<30 μg/mL), but viability significantly decreased at higher particle concentrations, suggesting a need for particle surface modification with poly (ethylene glycol) (PEG) for improved biocompatibility. The presence of PEG on particles after dialysis was indicated by an increase in size, an increase in zeta potential towards neutral, and spectroscopy results. Cell viability was improved in the absence of irradiation when cells were exposed to PEG-coated, dye-linked ZnO particles compared to non-surface modified particles. The present study shows that there is potential for biological application of dye-linked ZnO particles in photodynamic therapy.

## 1. Introduction

Reactive oxygen species (ROS) can oxidize DNA, proteins, and lipids, causing oxidative stress which can lead to permanent cellular damage [1,2,3]. While ROS are produced by cells under normal physiological conditions as byproducts during electron transport, excess ROS react with critical cellular components and can lead to cell death [4,5,6]. Photodynamic therapy (PDT) requires the production of these cytotoxic reactive species after activation of the photosensitizer by wavelength-matched light to initiate cell killing [7,8,9]. After excitement of the photosensitizer upon light irradiation, electrons return to the ground state and release the excess energy, which reacts with available molecular oxygen, generating ROS such as singlet oxygen (^1^O_2_), hydrogen peroxide, or superoxide anions [10,11,12,13]. PDT can be harnessed to kill unwanted cells, such as those in cancerous tumors. ROS are additionally of high interest in studies involving nanoparticles (NPs) because the production of ROS by cells in the presence of NPs is recognized as a prominent mechanism for nanotoxicity [14,15].

Zinc oxide (ZnO) can be synthesized into a variety of nanostructures, including NPs, nanotubes, nanorods, nanowires, and nanobelts [16,17,18,19,20,21,22,23,24,25]. These nanostructures are currently being studied for many applications, including use as sensors, optical devices, nanogenerators, and transducers due to their distinctive semiconducting, piezoelectric, and optical properties [13,26,27,28,29]. ZnO NPs are also being used in several biomedical applications, including bioimaging, drug delivery, and gene delivery [30,31,32,33,34,35].

Recently, organic dyes were linked to ZnO NPs to improve the photo and thermal stability of the dyes [36]. Dye-847-ZnO NPs were made by conjugating an organic cyanine dye, Dye 847 (Crysta-Lyn Chemical Company) to ZnO by an electrochemical oxidation technique of zinc metal as was previously described in Skorenko et al. [36]. The dye was bonded to ZnO through polar covalent linkages and the linkage is formed between C, O, and Zn in the presence of carboxylic acid, resulting in a metal-to-dye bridge [36]. The particles can be designed with a wide range of absorption peaks, including in the ultraviolet (UV), visible, and near-infrared (NIR) ranges by changing the conjugated dye during particle synthesis [36,37]. Dye 847 is an organic cyanine chromophore that absorbs NIR light with a peak maximum at 847 nm.

Current clinical PDT photosensitizers largely function in combination with visible light, which penetrates only millimeters into tissue, limiting the utility of this technique to superficial conditions such as skin cancer [12,38,39,40]. These novel Dye 847-ZnO NPs hold potential as photosensitizers for PDT at greater tissue depths because they have an excellent chemical stability and absorb light in the NIR range instead of the visible spectrum. Therefore, the potential of Dye 847-ZnO NPs to produce ROS in the absence of light exposure is relevant for biocompatibility, while the production of ROS in the presence of NIR light is relevant towards the use of Dye 847-ZnO particles as photosensitizers in PDT.

For biomedical applications, it is critical for NPs to be chemically and colloidally stable as well as biocompatible [19,22]. While ZnO NPs are generally understood to be cytotoxic [41,42,43,44,45,46], surface modification may limit this toxicity [47,48,49,50,51,52,53] and targeting of the particles may allow for directed cell killing while leaving non-targeted cells unharmed. ZnO particles linked to Dye 847 (Dye 847-ZnO NPs) were characterized and surface-modified by coating with poly (ethylene glycol) (PEG) to increase particle biocompatibility and reduce aggregation [54,55,56,57,58]. Particles were screened in this study for their toxicity in vitro before and after exposure to NIR light in human endothelial cells, which form the primary barrier for entry of NPs from the bloodstream to the body’s tissues, and in human breast carcinoma cells. The biocompatibility of the particles and release of ROS from the particles, with relevance towards their use in biomedical applications in PDT, was explored here.

## 2. Results

In order to explore the potential of organic dye-linked ZnO NPs for biomedical applications, the physicochemical and colloidal properties of the particles were explored before and after surface attachment of PEG. Important for their application in PDT, the level of ROS produced by the particles and toxicity towards cancerous and non-cancerous cells upon exposure to NIR light were studied, as detailed below. To decrease aggregation, a range of sonication times were tested (Supplement Section S5.1), and subsequent testing and particle use was done after 2 h of sonication of the stock solution and 4 h of sonication of the diluted working suspension (Table S1). Particle size was measured using several techniques both before and after surface modification with PEG, and Fourier transform infrared spectroscopy (FTIR) was used for the chemical analysis. To detect the generation of ROS by particles, the nitro blue tetrazolium salt (NBT) assay and 2′,7′-dichlorodihydrofluorescein diacetate (DCFDA) oxidation assay were used before and after exposure to a 850 nm light-emitting diode (LED) lamp light. The DCFDA assay results were also used to find the illumination time required to produce the highest level of ROS. The viability of human umbilical vein endothelial cells (HUVEC) and MCF7 cells were measured in the presence and absence of 850 nm light after treatment with PEG-Dye 847-ZnO NPs using cell counting kit-8 (CCK-8).

### 2.1. Cell Viability

**Figure 1:**
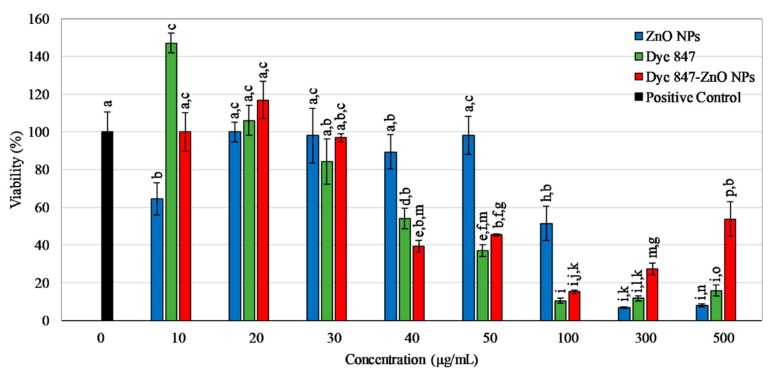
Viability of HUVEC after 24 h exposure to ZnO particles, Dye 847, or Dye 847-ZnO particles as a percentage of positive control (0 μg/mL, cells in EGM-2). Data shown are mean +/− standard deviation. Data were analyzed using analysis of variance (ANOVA) and post hoc t-test; concentration data that do not share any letters represent a statistically significant difference, *n* = 4, *p* < 0.05.

Confluent HUVEC were exposed to ZnO NPs, Dye 847-ZnO NPs, or Dye 847 alone at varying concentrations (10, 20, 30, 40, 50, 100, 300 and 500 μg/mL) for 24 h to test their cytotoxicity (Figure 1). HUVEC viability in the presence of ZnO NPs was concentration-dependent, with significant losses in viability above 100 μg/mL. Dye 847 alone exhibited cytotoxicity above 40 μg/mL, while Dye 847-linked ZnO particles caused a gradual decrease in cell viability starting at 40 μg/mL. The biggest drop in viability for these dye-linked NPs occurred at 100 μg/mL, followed by sudden increases thereafter from 300 to 500 μg/mL, which may be due to aggregation of the NPs that resulted in a limited uptake of large aggregates into cells. Since the viability of HUVEC showed minimal change upon exposure to lower concentrations (10, 20, and 30 μg/mL) of Dye 847-ZnO NPs but decreased as the concentration increased, particle surface modification with the hydrophilic polymer PEG was hypothesized to increase Dye 847-ZnO particle biocompatibility.

### 2.2. PEGylation

The molecular weight of PEG generally used to coat particles is in the range of 3400–10,000 Da. Satisfying the need for PEG to create a hydrophilic, biocompatible coating while avoiding the mushroom conformation that is more common with longer PEG chains [58,59,60,61,62] was the motivation for the use of PEG6000 in these studies. PEG was adsorbed on the particle surface for 12 h in an effort to make the particles more biocompatible [62,63,64,65]. To remove excess, un-adsorbed PEG, two methods were tested: centrifugation for 2 h at 1800× *g* and 24 °C and dialysis. Particle samples from the same stock that had been sonicated for 2 h and coated with PEG for 12 h were aliquoted in triplicate into three groups: 2 min vortexing, 15 min sonication, or 30 min sonication directly before analysis by dynamic light scattering (DLS). Samples were also analyzed by scanning electron microscopy (SEM) and FTIR. Readings from these techniques were taken at various points in particle synthesis: before addition of PEG, after 12 h of mixing with PEG, and after centrifugation or dialysis.

#### 2.2.1. Dynamic Light Scattering

The hydrodynamic size, polydispersity index, and the zeta potential of the samples were measured using dynamic light scattering (DLS) (Table 1). The negative charge on the surface of the NPs was likely due to the structure of the surface-conjugated dye, which was previously discussed in Skorenko et al. [36]. After coating, the slight increase towards neutral was a strong indicator of the PEG layer being present [56]. However, the downward shift in size from 346.5 ± 147.8 to 292.9 ± 98.2 nm and negative shift in zeta potential from −36.8 ± 19.8 to −53.16 ± 11.5 mV indicated that the PEG layer was likely removed from the nanoparticle surface during centrifugation. Hence, dialysis was tested in place of centrifugation; the size and zeta potential did not decrease as drastically after dialysis as compared to centrifugation, suggesting that the PEG layer remained present on the NPs.

#### 2.2.2. Scanning Electron Microscopy

**Figure 2:**
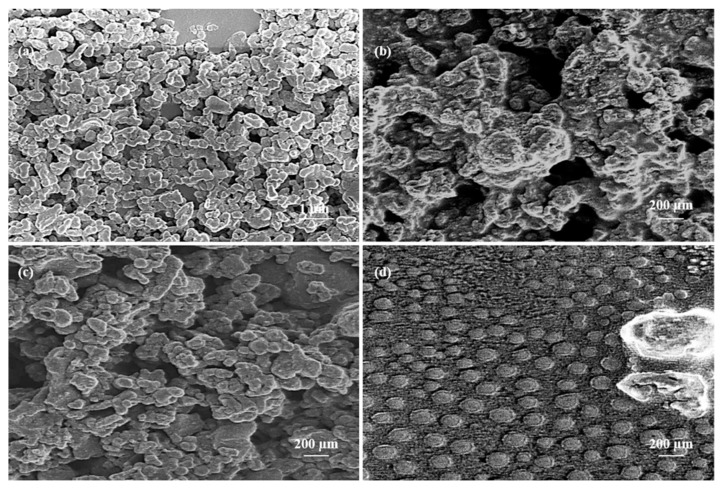
SEM images of Dye 847-ZnO NPs (**a**,**c**), Dye 847-ZnO NPs after addition of PEG followed by centrifugation (**b**), Dye 847-ZnO NPs after addition of PEG followed by dialysis (**d**).

SEM images of the NPs before the addition of PEG (Figure 2a,c) show that these particles do not have a defined spherical shape. The size of the single particles was measured using ImageJ software (Version number 1.49v). The average size of the dry particles before the addition of PEG measured using SEM was 150 ± 30 nm (*n* = 50 particles from 4 images), which was smaller than the hydrodynamic size measured using DLS due to the absence of bound water and likely aggregation. After coating with PEG followed by centrifugation, individual particles could not be identified via SEM (Figure 2b); the particles underwent significant agglomeration. After dialysis, the average size was found to be 170 ± 20 nm (*n* = 50 particles from 4 images), and less aggregation was present (Figure 2d). Due to the results obtained through SEM and DLS, we concluded that centrifugation was not the appropriate method for the removal of excess PEG for these particles, so dialysis was used in all subsequent studies.

#### 2.2.3. Fourier Transform Infrared Spectroscopy

**Figure 3:**
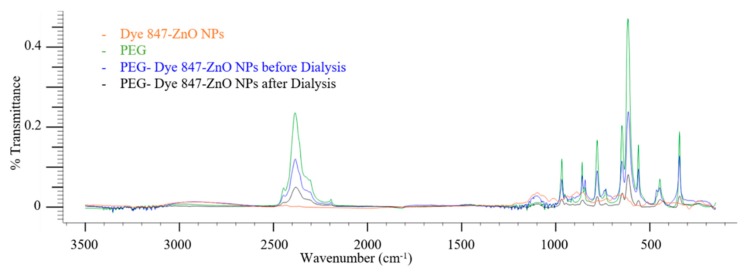
FTIR spectra (% Transmittance vs. wavenumber (cm^−1^)) of Dye 847-ZnO NPs without PEG (Dye 847-ZnO), PEG 6000 (PEG), PEG-Dye 847-ZnO NPs before dialysis, and PEG-Dye 847-ZnO NPs after dialysis.

Figure 3 shows four FTIR spectra from Dye 847-ZnO NPs, PEG 6000, PEG-coated Dye 847-ZnO NPs before dialysis, and PEG-coated Dye 847-ZnO NPs after dialysis. The strong broad bands at 3300–3250 and 1350–1260 cm^−1^ and bands at 2500–2250, 780–770 and 670–620 cm^−1^ correspond to the presence of alcohol and carbonyl groups in the PEG spectra. These bands were also observed in PEG-coated Dye 847-ZnO NPs before dialysis and PEG coated Dye 847-ZnO NPs after dialysis, indicating the presence of PEG before and after dialysis.

### 2.3. Production of ROS

The production of ROS by dye 847-ZnO NPs was measured using NBT and DCFDA assay. The NBT assay is a colorimetric assay for ROS production in which water-soluble NBT salt is converted to water-insoluble, blue NBT-diformazan in the presence of ROS [65,66,67,68]. DCFDA is commonly used to detect ROS in cells by oxidizing the non-fluorescent molecule to fluorescent 2′,7′-dichlorofluorescein (DCF); the amount of which is directly proportional to the ROS present [67,68,69]. The light source used in this study to induce ROS production from the Dye 847-ZnO NPs was a NIR LED lamp (λ = 850 nm, Power density = 4.6 mW/cm^2^).

**Figure 4:**
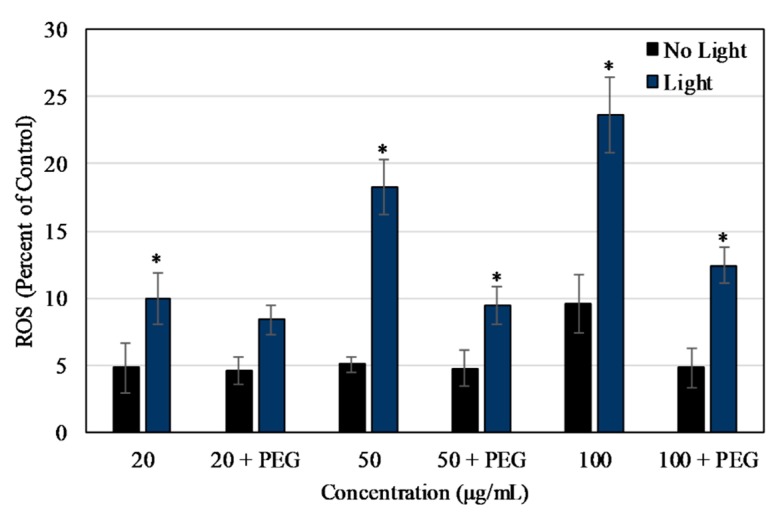
ROS produced by Dye 847-ZnO particles as a percentage of positive control (2% H_2_O_2_ in DI water). ROS measured using DCFDA 4 h after exposure to irradiation (2 min NIR lamp) or no light. Data shown are mean +/− standard deviation. Data were analyzed using ANOVA and post hoc t-test; data with ‘*’ represent a statistically significant difference between light and no light conditions at the stated concentration, *n* = 4, *p* < 0.05.

**Figure 5:**
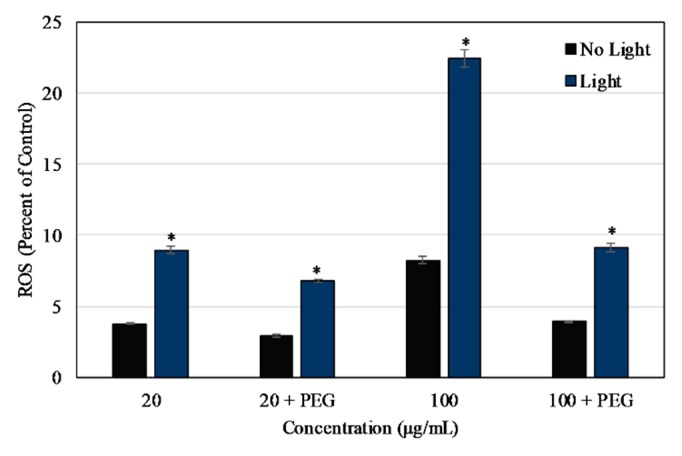
ROS produced by Dye 847-ZnO particles with or without PEG as a percentage of positive control (2% HX and 0.7% XOD in DI water). ROS measured using NBT 4 h after exposure to irradiation (2 min NIR lamp) or no light. Data shown are mean +/− standard deviation. Data were analyzed using ANOVA and post hoc t-test; data with ‘*’ represent statistically significant difference between light and no light conditions at the stated concentration, *n* = 4, *p* < 0.05.

As shown in Figure 4, the ROS released after NIR irradiation, measured at 4 h post-irradiation, was significantly higher in samples exposed to 2 min of NIR light. The level of ROS being released increased with increasing concentration and increased over time after irradiation (measured every 1 h for 8 h straight, Figure S1). The results were verified by conducting the NBT assay as shown in Figure 5, where the level of ROS released at 4 h post-NIR irradiation increased with increasing concentration. Values were normalized to the positive control, 2% H_2_O_2_, in both the assays. The release of ROS was partially negated by the presence of PEG in the NBT results.

### 2.4. Light Irradiation Time

**Figure 6:**
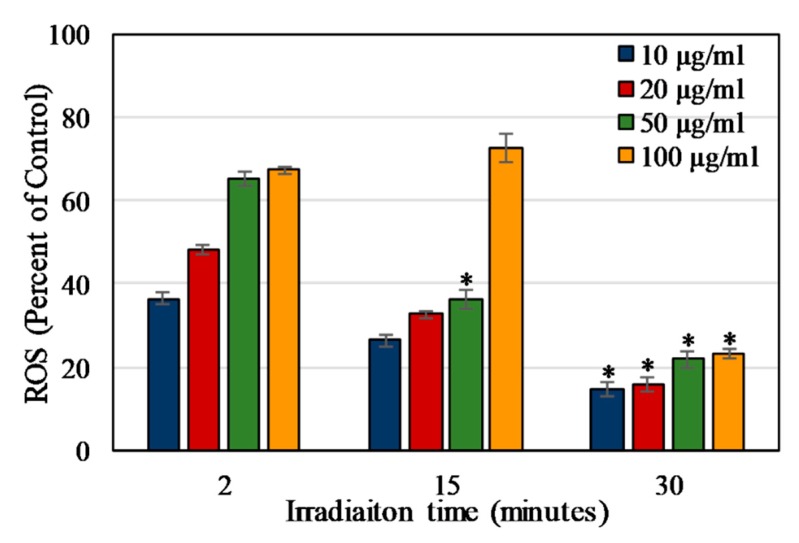
ROS produced by Dye 847-ZnO particles as a percentage of positive control (2% H_2_O_2_ in DI water). ROS measured using DCFDA 8 h after exposure to irradiation (2 min NIR lamp. Data shown are mean +/− standard deviation. Data were analyzed using ANOVA and post hoc t-test; data with ‘*’ represent statistically significant difference with respect to measurements at the same concentration but different irradiation times, *n* = 4, *p* < 0.05.

The DCFDA assay results were also used to find the total irradiation time required to produce the highest level of released ROS. The three different irradiation times used were 2, 15, and 30 min. As shown in Figure 6, the maximum ROS levels recorded after 2 and 15 min of irradiation on 100 μg/mL Dye 847-ZnO NPs were 67.21% ± 1.009% and 72.5% ± 3.454%, respectively, compared to the control hydrogen peroxide. The ROS level for 100 ug/mL dye 847-ZnO NPs at 15 min was higher than the same concentration at 2 min. The ROS levels for all other concentrations measured were greater at 2 than 15 min. The ROS level recorded after 30 min of exposure was also concentration-dependent, but the maximum ROS produced was 23.08% ± 1.130% which was significantly lower than the other two tested irradiation times. Therefore, subsequent studies were done with 2 min NIR irradiation as the extended light exposures may have resulted in breakdown of the DCF reporter molecule, complicating the interpretation of results. The DCFDA assay results after 2 min of NIR illumination were also plotted as percentage change in DCFDA over time, which showed the highest rate of change in the first 3 h, after which the rate of change became near-constant (Figure S2). Therefore, the reaction time for the DCFDA assays was chosen to be 3 h for cellular studies.

### 2.5. Detection of ROS in Cells Exposed to Dye 847-ZnO Particles

After testing the potential of Dye 847-ZnO NPs to produce ROS, it was important to check the ROS production in vitro. The ROS produced by Dye 847-ZnO NPs exposed to 850 nm light in non-cancerous HUVEC and cancerous MCF7 in vitro cells was measured using the DCFDA assay.

**Figure 7:**
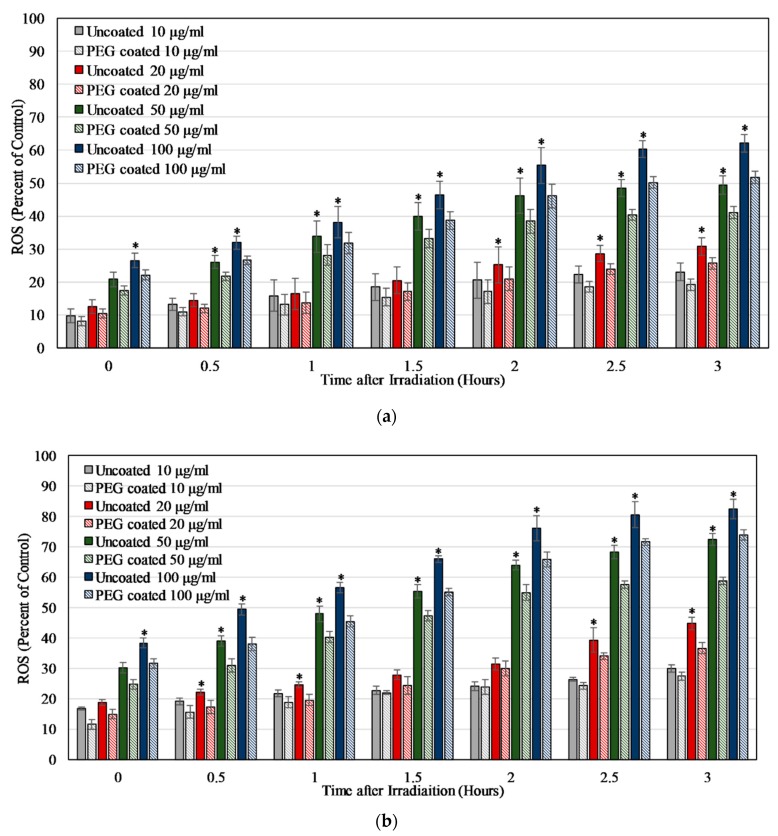
ROS produced as a percentage of positive control (2% H_2_O_2_ in cell media) after HUVEC (**a**) or MCF7 (**b**) were exposed to uncoated and PEG-coated Dye 847-ZnO particles measured using DCFDA after exposure to irradiation (2 min NIR lamp). Data shown are mean +/− standard deviation. Data were analyzed using ANOVA and post hoc t- test; uncoated particle data with ‘*’ represent statistically significant difference with respect to PEG-coated particle, concentration-matched data, *n* = 4, *p* < 0.05.

As shown in Figure 7a,b, the ROS produced when HUVEC and MCF7 cells were exposed to Dye 847-ZnO NPs and light was concentration-dependent. This concentration dependent increase in ROS level stabilized around 3 h, with further changes in ROS being statistically insignificant. The ROS production was partially negated by the presence of PEG in both the cell lines. For HUVEC, the ROS produced after 3 h of exposure to 100 μg/mL of uncoated and PEG-coated NPs was 62.10% ± 2.62% and 51.75% ± 1.75%, respectively, compared to 2% H_2_O_2_ in cell media. In comparison, at 3 h MCF7 cells samples contained 82.64% ± 3.17% and 73.93 ± 1.73% ROS compared to H_2_O_2_ upon exposure to 100 μg/mL uncoated and PEG-coated particles.

### 2.6. In Vitro Toxicity on EXPOSURE to PHOTOSENSITIZER NPs and Light

To assess the extent of cell damage caused due to the ROS produced by Dye 847-ZnO NPs, the cell viability of both HUVEC and MCF7 cells was measured using CCK-8 after nanoparticle exposure and NIR irradiation.

**Figure 8:**
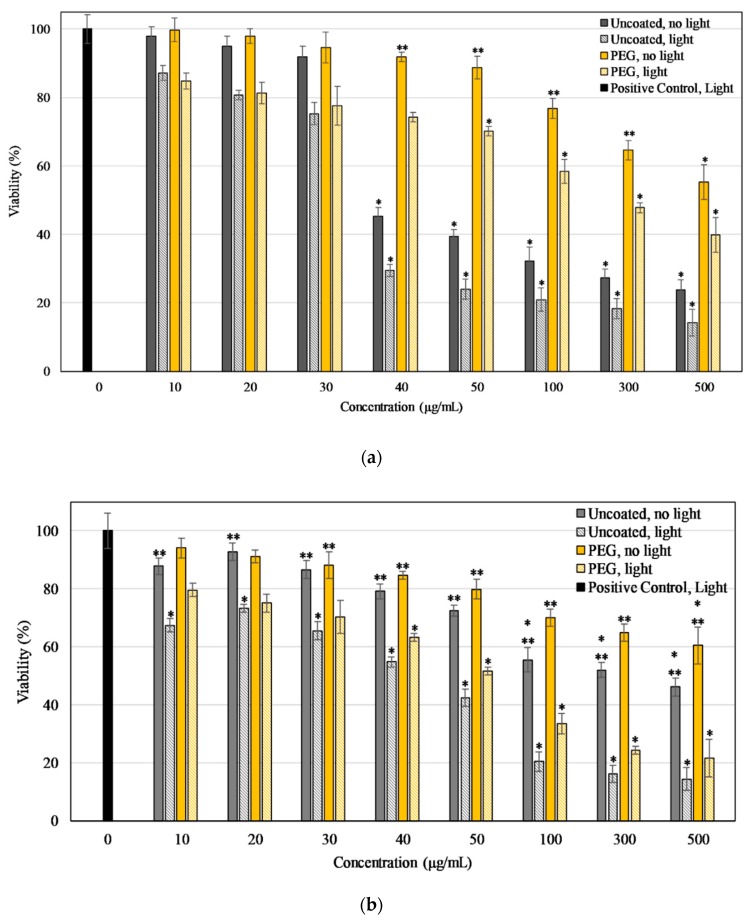
Viability of HUVEC (**a**) and MCF7 (**b**) after 24-h exposure to uncoated and PEG coated Dye 847-ZnO NPs at various concentrations as a percentage of positive control (0 μg/mL, cells in EGM-2/MEM*) after exposure to irradiation (2 min NIR lamp). Data shown are mean +/− standard deviation. Data were analyzed using ANOVA and post hoc t-test; ‘*’ represent samples that are statistically significant with respect to control (cells without NPs) and for uncoated and PEG-coated pairs in concentration matched conditions ‘**’ represent a statistically significant difference between light and no light data, *n* = 4, *p* < 0.05.

As shown in Figure 8a, the results indicate that concentrations of PEG-Dye 847-ZnO particles had no statistically significant effect on cell viability up to 50 μg/mL in the absence of 850 nm light, in contrast to the uncoated particles that affected cell viability at 40 μg/mL. Viability significantly decreased at 500 μg/mL to 23.73% ± 2.98% in response to uncoated NPs, whereas the viability reduced to only 55.31% ± 5.06% for PEG-coated NPs, indicating the expected improved biocompatibility of PEG-coated particles.

As shown in Figure 8b, MCF-7 viability in the presence of uncoated ZnO-Dye 847 NPs was concentration-dependent for cells exposed to particles, with or without irradiation. The exposure to these NPs for 24 h reduced the viability in a concentration-dependent manner, to an extreme of 46.09% ± 3.00% at 500 μg/mL, the highest concentration used. Irradiation with light significantly increased particle-related cytotoxicity with a viability of MCF7 cells reduced to 42.43% ± 2.94% at a concentration of only 50 μg/mL and to 14.29% ± 3.94% at 500 μg/mL.

## 3. Discussion

Concentrated ZnO nanoparticles conjugated with Dye 847 aggregated in water and precipitation was seen by eye. Aggregation in the samples was undesirable since particle properties vary greatly with aggregation and the interactions between the nanoparticles and the cells are size-dependent. Determination of the sonication time necessary to increase the proportion of individual as opposed to aggregated particles was optimized. Two hours of sonication of the stock solution plus 4 h of sonication of the diluted sample was used subsequently for all nanoparticle samples. Despite these efforts, in this study, HUVEC viability in the presence of the tested nanoparticles revealed concentration-dependent interactions, with cell viability dropping at moderate concentrations and then increasing at higher concentrations. This increase at high concentrations is likely due to the aggregation of nanoparticles, as larger aggregates are less likely to interact with cells [56]. Surface modification with PEG was hypothesized to decrease aggregation and increase the biocompatibility of the Dye 847-ZnO nanoparticles.

The presence of the PEG coating was confirmed by comparing the hydrodynamic size, poly dispersity index, and the zeta potential of the samples before and after PEGylation. The hydrodynamic size of the nanoparticles after coating with PEG increased in comparison to uncoated particles. Particle zeta potential changed from approximately −50 to around −30 mV after the 12 h incubation in PEG solution, which is an additional strong indicator of the adsorption of the neutral polymer to the particle surface [70]. However, the hydrodynamic size was reduced after centrifugation and zeta potential became more negative (Table 1), likely indicating that centrifugation removed the PEG from the particle surface. Conversely, the same changes in size and zeta potential were not seen when excess PEG was removed from the sample through dialysis, suggesting that the PEG layer remained intact on the nanoparticles. Zeta potential measurements indicated a high colloid stability (greater than |25 mV| [71]) as the PEG-coated particles were −35.9 ± 9.6 and −31.3 ± 6.9 mV before and after dialysis, respectively. The negative charge on the surface of the nanoparticle is likely due to the presence of the carboxylic acid on dye 847 [36]. Finally, the presence of PEG at the surface of Dye 847-ZnO nanoparticles was verified using FTIR.

In ROS studies, the uncoated nanoparticles were capable of producing ROS upon irradiation with 850 nm NIR light, as measured via DCFDA. The NBT assay was used to verify these results and the assay also showed that the release of ROS was partially negated by the presence of PEG on the particle surface, which is likely due to a decrease in the nanoparticle surface availability for catalytic reactions. In other words, this decrease in ROS is potentially due to the hydrophilic PEG layer reducing direct NP interactions with the surrounding chemical species that are required for the formation of ROS [72]. The NIR light exposure time was chosen to be 2 min as the ROS level produced at 15 and 30 min was significantly lower. This effect might have been related to the particles or the assay since the reporter molecule was less effective after the longer exposure times to NIR light. On exposure to Dye 847-ZnO NPs and light, ROS results in vitro for HUVEC and MCF7 depicted a particle concentration-dependent increase in ROS detected. ROS production differed in vitro between uncoated and PEG-coated NPs in both cell types, with PEG negatively impacting ROS production, as seen in several other studies [50,73,74,75]. Additionally, ROS produced in MCF7 cultures was significantly higher than HUVEC in our study.

In cellular studies with PEG-coated nanoparticles, the cell viability of HUVEC remained unchanged up to 50 μg/mL, which was a higher concentration threshold than with uncoated nanoparticles, indicating the improved biocompatibility of PEG- coated particles. The cell killing in the presence of uncoated particles after light irradiation was higher than the PEG-coated particles for both the cells lines. These same data plotted as the percent difference in the irradiated values compared to the no light concentration-matched samples (Figure S3) indicate that at all tested concentrations, particles exposed to NIR light were more cytotoxic than the no light controls. Additionally, higher concentrations of particles lead to higher proportions of light-related cell killing. Furthermore, at all concentrations for MCF7 cells and concentrations above 40 μg/mL for HUVEC, uncoated particles caused a significantly higher proportion of killing (irradiated compared to no light) than PEG-coated samples. These data indicate that while NIR-irradiated PEG-coated particles do exhibit cytotoxicity, the overall effect is slightly negated by the presence of PEG. Importantly, these data pair with the ROS results and show that the cell damage done by PEG-Dye 847-ZnO NPs was significantly more pronounced in MCF7 cells than HUVEC. These results indicate that Dye 847-ZnO NPs exhibited preferential cytotoxicity. The differences in lysosomal capacity and mitochondrial activity between cancerous and non-cancerous cells are additional factors that have been shown to contribute to preferential toxicity [76,77,78]. Similar results were observed in a study in which ZnO nanoparticles showed higher toxicity towards the cancer cells and minimal toxicity towards non-cancerous cells using human lung adenocarcinoma A549, human hepatocellular carcinoma HepG2, and human bronchial epithelial BEAS-2B, and two primary rat cells. The mechanism of toxicity was found to be selective apoptosis, which was facilitated by reactive oxygen species (ROS) [79]. Furthermore, in a separate study, a 30% increase in apoptosis and an 80% increase in necrosis were induced in HeLa cells by ZnO flower-like nanostructures, whereas the effect on the non-cancerous murine fibroblast (L929) cells was only 5–10% for both apoptosis and necrosis [80]. The mechanism for the preferential cytotoxicity of ZnO towards cancer cells is currently not well understood, but ZnO nanoparticles hold potential in several biomedical applications for cell killing due to their toxic effects to cancers and bacteria [56,57,81,82].

After ROS studies, the oxidative species quantum yield of the particles upon NIR irradiation with power density 4.6 mW/cm^2^ was calculated to check if the efficiency was comparable with studies using light sources with power density between 25 and 600 mW [83,84,85,86,87]. The quantum yield of Dye 847, Dye 847-ZnO, and PEG-Dye 847-ZnO NPs at 850 nm were calculated using the method formulated by Clement et al. [88]. The quantum yield (η) values were calculated in comparison with Indocyanine Green (ICG, Fisher Scientific, Hampton, NH, USA), a widely used photosensitizer with absorbance in the NIR range with reported η ranging from 0.0008 to 0.40 [89,90,91,92,93,94,95]. The η for 20 and 100 μg/mL Dye 847-ZnO were calculated to be 0.40 and 0.32, respectively, whereas the η for 20 and 100 μg/mL PEG Dye 847-ZnO were calculated to be 0.68 and 0.60, respectively (Table S2). These values were comparatively higher than the η of the other available photosensitizers. These are similar to ROS assay results, as the ROS levels increased with increasing concentrations and were slightly suppressed due to the presence of PEG. The η for Dye 847-ZnO NPs ranged from 0.32 to 0.68 for different concentrations of uncoated and PEG-coated particles. The η of Dye 847 was calculated to be 0.35 and 0.50 for 20 and 100 μg/mL, respectively. The absorbance spectra of Dye 847, Dye 847-ZnO, and PEG Dye 847-ZnO NPs were very similar, indicating that the binding of Dye 847 to ZnO did not affect its absorption properties (Figure S4).

Due to limited toxicity when coated with PEG, the ability to produce ROS upon NIR irradiation, preferential cytotoxicity, and high η using a NIR lamp with low power density, these novel Dye 847-ZnO NPS hold promise to act as photosensitizers for biomedical applications in PDT.

## 4. Materials and Methods

### 4.1. Cell Viability Assay

Confluent HUVEC grown on a well plate coated with 8 µg/mL collagen (Gibco, Gaithersburg, MD, USA) were exposed to NPs at different concentrations (10, 20, 30, 40, 50, 100, 300 and 500 μg/mL) for 24 h. Cell Counting Kit-8 (CCK- 8 kit, Sigma, St. Louis, MO, USA) was used to measure the viability of cells. CCK-8 allows sensitive colorimetric determination of the number of viable cells using a formazan dye. After incubation, absorbance of the formazan dye was recorded at 450 nm using a microplate reader (Biotek Synergy H1, Winooski, VT, USA). Tested samples included ZnO NPs, Dye 847 alone, as well as ZnO particles conjugated to dye 847. Stock solutions of the NPs were prepared by sonicating and vortexing the NPs in water at a concentration of 1 mg/mL. Once the stock solution was prepared, particles were sonicated again for the time determined in the previous test and diluted to working concentrations in endothelial growth medium-2 (EGM-2, Lonza, Walkersville, MD, USA). The concentrations of NPs represent physiologically relevant doses of NPs, based on previous studies [41,43,44,46,96,97,98]. Cells in EGM-2 without NPs served as the positive control, and cells exposed to 2% Triton X-100 (Sigma- Aldrich, St. Louis, MO, USA) in EGM-2 served as the negative control.

### 4.2. PEGylation

After optimization of sonication time, PEG (6000 MW, Alfa Aesar, Haverhill, MA, USA) was adsorbed to the surface of Dye 847-ZnO NPs. PEG is biocompatible, increases colloidal stability, and increases circulation time in vivo [56,58,62,99,100,101,102,103,104,105,106]. Particle samples from the same stock that had been sonicated for 2 h were sonicated for 6 h (including 2 h of stock sonication), and 1.05 × 10^−3^ M PEG solution was added to the nanoparticle stock (1 mg/mL). The solution was stirred continuously for 12 h to complete the adsorptive PEGylation process.

For some samples, excess PEG was removed by centrifugation for 2 h at 1800× *g* and 24 °C, after which the supernatant was removed and particles were re-suspended. Alternatively, excess PEG was removed via dialysis (Slide- A- Lyzer Dialysis cassette with 20 kDa cutoff, Thermo Scientific, Waltham, MA, USA). Both methods were tested to determine the most effective means of removing excess PEG while maintaining particle colloidal stability.

### 4.3. Particle Characterization

Particles were analyzed by DLS, scanning electron microscopy (SEM, Supra 55 VP, Zeiss, NY, USA), and Fourier transform infrared spectroscopy (FTIR, 190-FTIR Spectrometer, Thermo Fisher Scientific, Waltham, MA, USA). Readings from these techniques were taken at various points in particle synthesis: before addition of PEG, after 12 h of mixing with PEG, and after centrifugation or dialysis. Size and zeta potential were measured by DLS at a nanoparticle concentration of 1.9 × 10^−2^ mg/mL in water. NPs in water were dried on a SEM sample tab and sputter coated before SEM imaging. For FTIR, a droplet of particles suspended in water was dried on a NaCl pellet and the spectrum was measured.

### 4.4. Production of ROS

#### 4.4.1. Using NBT Oxidation Assay

Varying concentrations (20, 50, and 100 μg/mL) of uncoated and PEG-coated Dye 847-ZnO NPs, 30 mM of hypoxanthine (HX, Research Products International Corp, Mt. Prospect, IL, USA) solution in 50 mM in potassium hydroxide (KOH, Amresco, Solon, OH, USA), xanthine oxidase (XOD from bovine milk, Sigma-Aldrich, St. Louis, MO, USA) solution diluted to 50% in PBS, and 3 mM of NBT solution in 70% *N*,*N*-Dimethylformamide (DMF, Sigma-Aldrich, St. Louis, MO, USA) were prepared. HX and XOD were used together to generate ROS to be used as control [99]. The nanoparticle and control samples were irradiated with the NIR source (λ = 850 nm, Power density = 4.6 mW/cm^2^, Amazon, Seattle, WA, USA) for 2 min with each concentration having 4 replicates. The nanoparticle samples were mixed with NBT in the volume ratio of 20 parts nanoparticle suspension: 2 parts NBT solution. HX and XOD solutions were mixed in the volume ratio of 2 parts HX: 0.7 parts XOD: 2 parts NBT: remaining parts DI water to serve as the positive control. The samples were incubated for 4 h, after which the absorbance was measured at 450 nm using a plate reader (Synergy H1, Biotek).

#### 4.4.2. DCFDA

Various concentrations (20, 50, and 100 μg/mL) of uncoated ZnO- Dye 847 NPs having 4 replicates each were irradiated with the NIR source for 2 min. Hydrogen peroxide (100 μL of 2%, H_2_O_2_, Fisher Scientific, Hampton, NH, USA) diluted in DI water served as the positive control and DI water was the negative control. DCFDA (100 μL of 20 mM, Sigma-Aldrich, St. Louis, MO, USA) diluted in PBS with magnesium and calcium was added to each well and incubated for 2 h. The fluorescence (*E_x_*/*E_m_* = 495/529 nm) of all samples was measured in a microplate reader (Spectra i3x, Molecular Devices, San Jose, CA, USA) every 1 h for 8 h.

### 4.5. Selection of Light Irradiation Time

The irradiation time required to produce the highest level of released ROS was investigated using DCFDA. Four replicates of uncoated ZnO-Dye 847 NPs (10, 20, 50, and 100 μg/mL) were irradiated with the NIR source for 2, 15, and 30 min. As used earlier, 2% H_2_O_2_ diluted in DI water served as the positive control and DI water served as the negative control. DCFDA diluted in PBS (100 μL of 20 mM) was added to each well and incubated for 2 h. The fluorescence (*E_x_*/*E_m_* = 495/529 nm) was measured in a microplate reader (Spectra i3x, Molecular Devices) every 1 h for 8 h.

### 4.6. Detection of ROS in HUVEC and MCF7 Cells Exposed to Dye 847-ZnO Particles

Confluent HUVEC grown on a 96-well plate coated with 8 μg/ mL collagen and MCF7 cells grown directly on a well plate were exposed to uncoated and PEG-coated ZnO-Dye 847 NPs at different concentrations (10, 20, 50, and 100 μg/mL) for 24 h, each concentration having 4 replicates. Samples were exposed to the NIR source for 2 min, after which 100 μL of 20 mM DCFDA diluted in PBS was added to each well and incubated for 2 h. The fluorescence (*E_x_*/*E_m_* = 495/529 nm) was measured in a microplate reader (Spectra i3x, Molecular Devices) every 15 min for 3 h. H_2_O_2_ (2%) diluted in EGM-2 and cells in EGM-2 served as the positive and negative controls, respectively, for HUVEC; 2% H_2_O_2_ diluted in Minimum Essential Medium supplemented with 0.01 mg/mL human recombinant insulin and 10% fetal bovine serum (MEM*, Fisher Scientific) and cells in MEM* served as controls for MCF7 cells.

### 4.7. In Vitro Toxicity on Exposure to Photosensitizer NPs and Light

Confluent cells were exposed to uncoated and PEG-coated ZnO-Dye 847 NPs at varying concentrations (10, 20, 50, and 100 μg/mL) for 24 h, each concentration having 4 replicates. Only one set of samples were exposed to NIR source for 2 min and compared to the other set of samples with no exposure to light. Cell Counting Kit-8 (CCK- 8 kit) was used to measure the viability of cells. After incubation, the absorbance of the formazan dye was recorded at 450 nm using a microplate reader (Spectra i3x, Molecular Devices). Cells in media without NPs served as the positive control, and cells exposed to 2% Triton X-100 in media served as the negative control.

### 4.8. Statistical Analysis

To determine whether statistically significant differences between samples existed, an ANOVA was performed on the results, which was followed by a post hoc t-test with Tukey’s correction to determine inter-sample significance.

## 5. Conclusions

These studies revealed that the toxicity of the dye-linked ZnO NPs was concentration-dependent. Cell viability decreased at moderate concentrations, suggesting a need for particle surface modifications. The presence of a PEG coating on the NPs was confirmed through the size measurements, electron microscopy, and FTIR. The cytotoxic effects and accumulation of NPs around the cells were reduced to a great extent with the help of the PEG coating. The present study has shown that there is potential for biological application of the dye-linked ZnO particles. Furthermore, that particles successfully released ROS in the presence of a NIR light source with as little as two minutes of irradiation, and cell viability dropped in response to this ROS release. These particles hold potential as photo-stable NIR photosensitizers for application in PDT.

## Figures and Tables

**Table 1:** Hydrodynamic size, PdI, and zeta potential of Dye 847-ZnO NPs with or without PEG and washed/collected via centrifugation or dialysis. Data shown are mean ± standard deviation, n = 3 independent samples read three times each by the instrument. Stock solution was sonicated for 2 h before listed sonication times before and after addition of PEG.

Sample		Diameter (nm)	PdI	Zeta Potential (mV)
No PEG	4 h sonication	418.6 ± 192	0.44	−47.8 ± 20
	6 h sonication	284.2 ± 128.6	0.41	−50.7 ± 15.2
With PEG	2 min vortex	751.5 ± 238.1	0.53	−41.1 ± 13.2
	15 min sonication	435.1 ± 169	0.47	−39.3 ± 18.8
	30 min sonication	346.5 ± 147.8	0.45	−36.8 ± 19.8
With PEG, Centrifugation	2 min vortex	819.57 ± 54.6	0.57	−50.5 ± 16.8
	15 min sonication	379.5 ± 142.7	0.38	−53.7 ± 11.4
	30 min sonication	292.9 ± 98.2	0.39	53.16 ± 11.5
With PEG, Dialysis	2 min vortex	675.2 ± 212.3	0.47	34.5 ± 7.9
	15 min sonication	365.2 ± 99.1	0.46	31.3 ± 6.9
	30 min sonication	217.8 ± 82.9	0.29	33.9 ± 9.5

## Supplementary Materials

The following are available online at https://www.mdpi.com/1996-1944/13/1/17/s1, Figure S1: ROS produced by Dye 847-ZnO particles without PEG at concentrations of 10, 20, 50, and 100 ug/mL, measured by DCFDA after three different light exposure times: (**a**) 2 min, (**b**) 15 min, and (**c**) 30 min and normalized to the positive control (2% H_2_O_2_ in DI water). Data shown are mean +/− standard deviation, Figure S2: Percent difference of ROS produced by Dye 847-ZnO particles. ROS measured using DCFDA every 1 h for 8 h straight after exposure to irradiation (2 min NIR lamp). Data shown are mean +/− standard deviation, Figure S3: Percent difference of viability of HUVEC (**a**) and MCF7 (**b**) after 24-h exposure to uncoated and PEG-coated Dye 847-ZnO NPs at various concentrations after exposure to irradiation (2 min NIR lamp), plotted as percentage difference between light and no light at each concentration. Data shown are mean +/− standard deviation, Figure S4: Absorbance spectra of (**a**) Dye 847- 100 μg/mL, (**b**) 100 μg/mL Dye 847-ZnO NPs, and (**c**) 100 μg/mL PEG coated Dye 847-ZnO NPs, Table S1: Hydrodynamic size, polydispersity index (PdI), and zeta potential of Dye 847-ZnO particles measured after 2 h of stock sonication and 2, 4, 6 and 8 h of sonication at the working concentration, Table S2: Calculated Quantum yield (η) of Dye 847, Dye 847-ZnO and PEG-Dye 847-ZnO NPs at different concentrations (20 and 100 μg/mL) after exposure to irradiation (2 min NIR lamp) by comparing with the quantum yield of a standard photosensitizer (Indocyanine Green (ICG, η = 0.40)).
