# Supplementary material for: Near Infrared-Activated Dye-Linked ZnO Nanoparticles Release Reactive Oxygen Species for Potential Use in Photodynamic Therapy
Source: Materials (Basel). 2019 Dec 18;13(1):17. doi: 10.3390/ma13010017 (PMC6982235; doi:10.3390/ma13010017)
Supplement: Supplementary file 1 [file materials-13-00017-s001.pdf]

materials

Supplementary

Near Infrared-Activated Dye-Linked ZnO Nanoparticles Release Reactive Oxygen Species for Potential Use in Photodynamic Therapy

Jaspreet Singh Nagi [1], Kenneth Skorenko [2], William Bernier [2,3], Wayne E. Jones [3,4] and Amber L. Doiron [1,*]

[1] Department of Electrical and Biomedical Engineering, University of Vermont, Burlington, VT 05405, USA; Jaspreet-Singh.Nagi@uvm.edu
[2] ChromaNanoTech LLC, Binghamton, NY 13902, USA; kenneth@chromananotech.com (K.S.); wbernier@chromananotech.com (W.B.)
[3] Department of Chemistry, Binghamton University (SUNY), Binghamton, NY 13902, USA; wayne.jones@unh.edu
[4] Provost and Vice President for Academic Affairs, University of New Hampshire, Durham, NH 03824, USA
* Correspondence: amber.doiron@uvm.edu; Tel.: +1-802-656-3382

Received: 14 October 2019; Accepted: 12 December 2019; Published: xxxxx

1. Supplementary Data

1.1. Determining Sonication Time for ZnO

Visual examination and preliminary studies with DLS (not shown) indicated that Dye 847-ZnO NPs aggregated in water. To overcome this challenge, Dye 847-ZnO NPs were sonicated (VWR bath sonicator, 20–50 kHz output range) for various periods of time. Subsequent to sonication, the size and zeta potential were measured by DLS (Malvern ZetaSizer ZS90, UK). A stock solution of Dye 847-ZnO NPs was prepared at a concentration of 1 mg/mL with 2 h of sonication. The stock was diluted at the ratio of 1:50 to achieve a working concentration of 20 µg/mL and sonicated for an additional 2, 4, or 6 h prior to measure the size and zeta potential in triplicate using DLS.

Table S1. Hydrodynamic size, polydispersity index (PdI), and zeta potential of Dye 847-ZnO particles measured after 2 h of stock sonication and 2, 4, 6 and 8 h of sonication at the working concentration.

Time	Diameter (nm)	PdI	Zeta Potential (mV)
2 h	284.6 ± 95.9	0.38	−46.13 ± 10.7
4 h	242.83 ± 77.2	0.34	−47.83 ± 12.1
6 h	498.1 ± 137.1	0.47	−51.73 ± 10.9
8 h	384.33 ± 67.4	0.55	−48.46 ± 10.4

The size reading obtained at 4 h was 242.83 ± 77.2 nm, which was the lowest size achieved in these tests. The zeta potential was fairly consistent throughout all the readings (Table S1). Therefore, subsequent testing and particle use was done after 2 h of sonication of the stock solution and 4 h of sonication of the diluted working suspension.

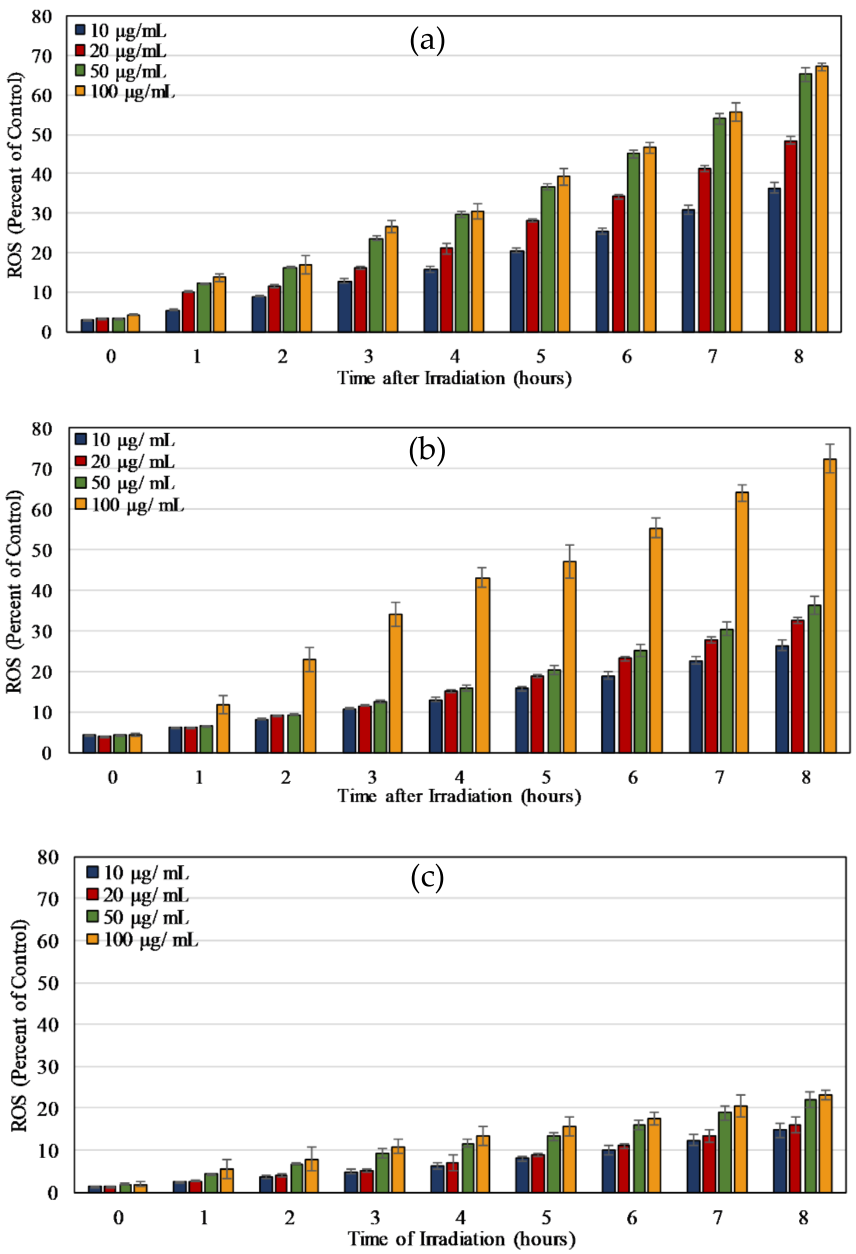

Figure S1. ROS produced by Dye 847-ZnO particles without PEG at concentrations of 10, 20, 50, and 100 ug/mL, measured by DCFDA after three different light exposure times: (**a**) 2 min, (**b**) 15 min, and (**c**) 30 min and normalized to the positive control (2% H_2O_2 in DI water). Data shown are mean +/− standard deviation.

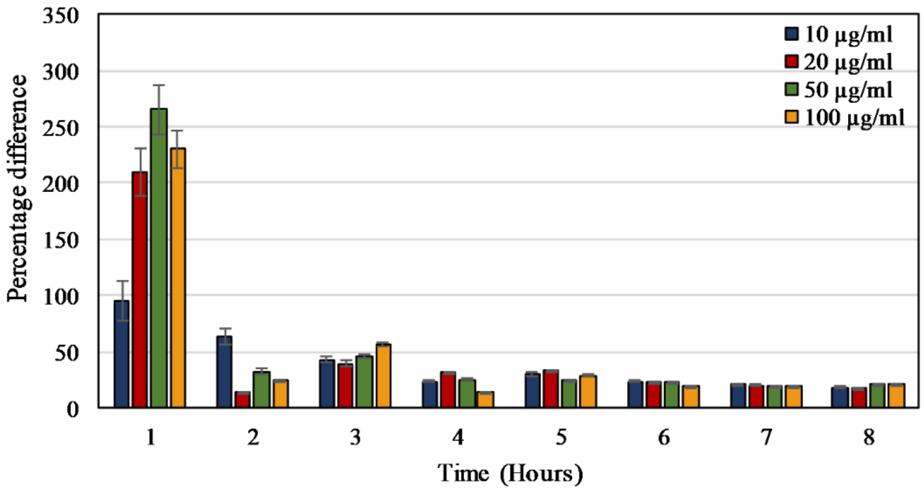

Figure S2. Percent difference of ROS produced by Dye 847-ZnO particles. ROS measured using DCFDA every 1 h for 8 h straight after exposure to irradiation (2 min NIR lamp). Data shown are mean +/− standard deviation.

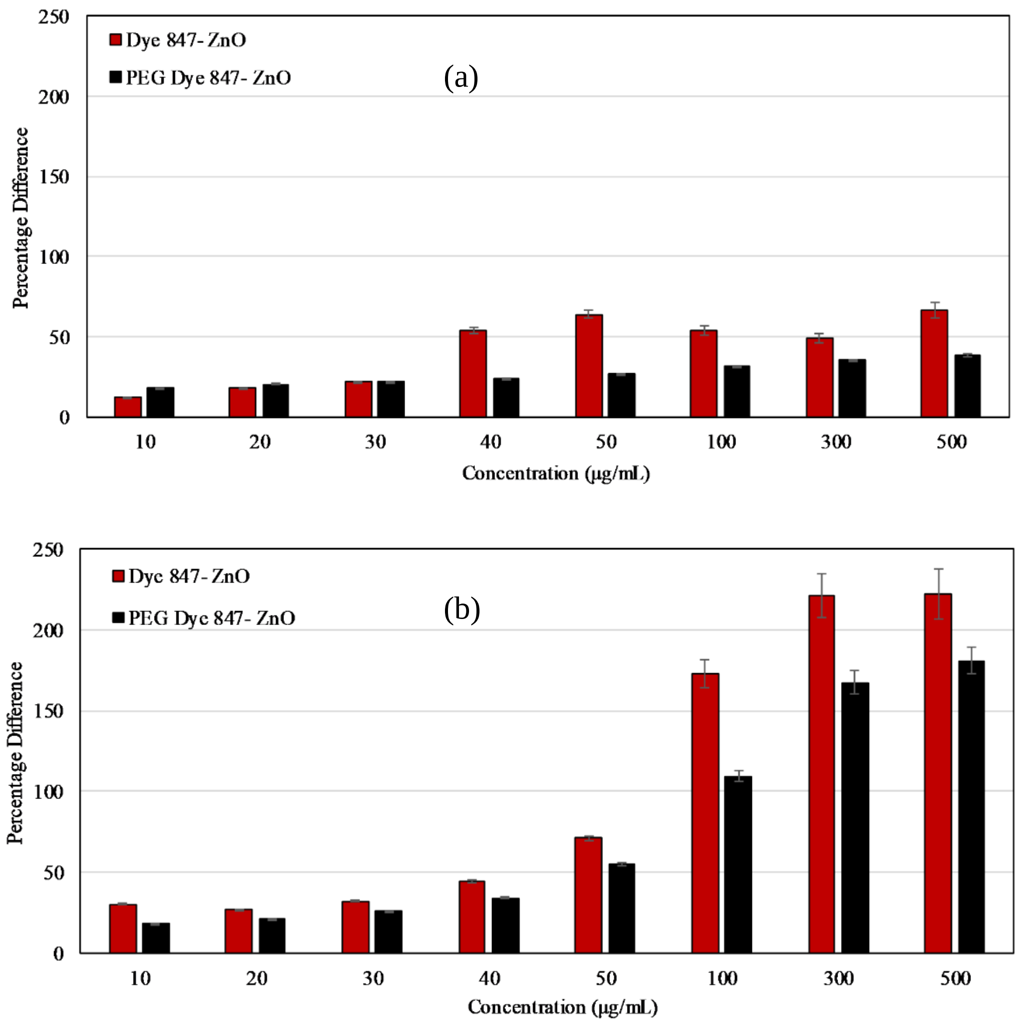

Figure S3. Percent difference of viability of HUVEC (**a**) and MCF7 (**b**) after 24-h exposure to uncoated and PEG-coated Dye 847-ZnO NPs at various concentrations after exposure to irradiation (2 min NIR lamp), plotted as percentage difference between light and no light at each concentration. Data shown are mean +/− standard deviation.

2.2. Absorbance

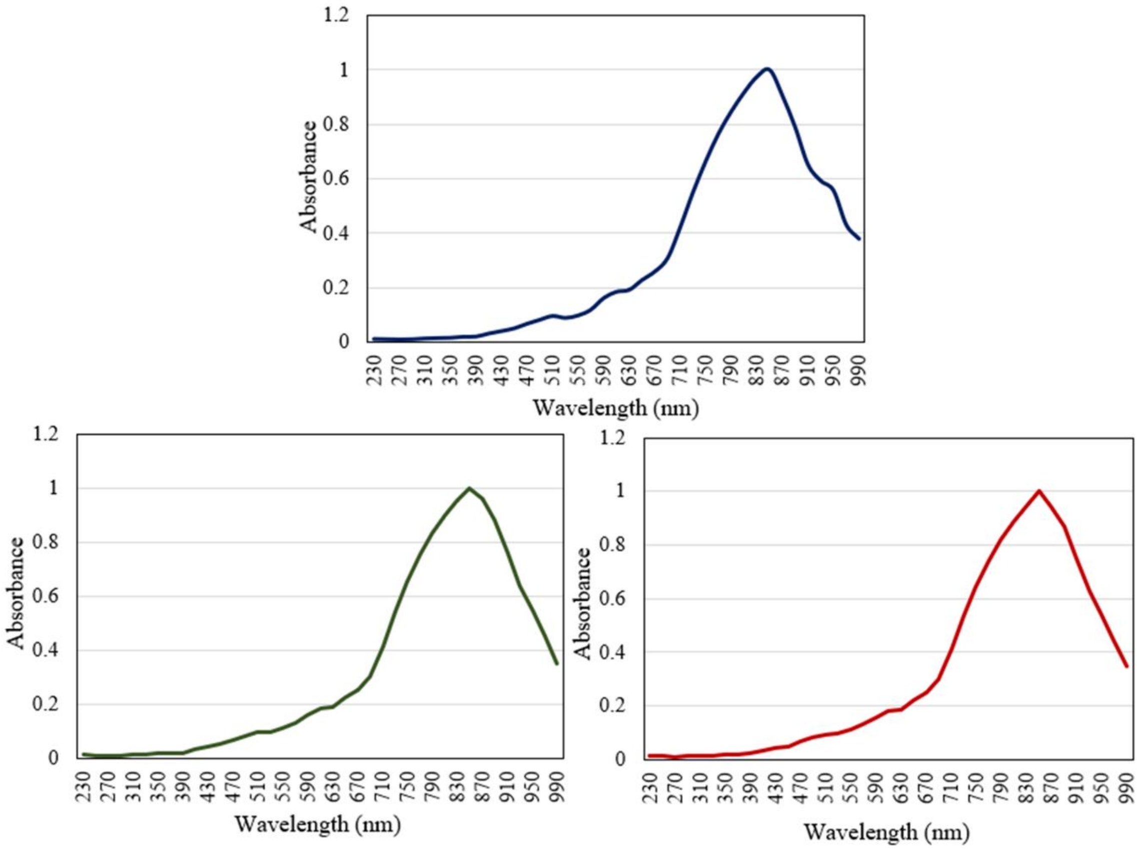

Figure S4. Absorbance spectra of (**a**) Dye 847–100 µg/mL, (**b**) 100 µg/mL Dye 847-ZnO NPs, and (**c**) 100 µg/mL PEG coated Dye 847-ZnO NPs.

1.3. Quantum Yield (η)

Oxidative species quantum yield of Dye 847, Dye 847-ZnO and PEG-Dye 847-ZnO NPs was calculated using the method shown by Clement et al. [92]. In this method, the number of photons emitted in time t were calculated using the formula:

$$E = n \times h \times \nu$$

where,

n = number of photons

h = Planck's constant

ν = frequency

And, Energy divided by Planck's constant gave us photons emitted per second, where energy was expressed in terms of E = P X t (P = Optical power of the lamp, t = irradiation time).

To attain the ROS levels of ICG, confluent MCF7 cells grown on a well plate were exposed to 100 µg/mL ICG for 24 h having 4 replicates. Samples were exposed to the NIR light source (λ = 780 nm, Power density = 69.33 mW/cm2) for 2, 15, and 30 min after which 100 µL of 20 mM DCFDA diluted in PBS was added to each well and incubated for 2 h. The fluorescence (Ex/Em = 495/529 nm) was measured in a microplate reader (Spectra i3x, Molecular Devices) every 15 min for 3 h.

Number of photons absorbed in time t by standard photosensitizer (ICG) were calculated using the formula:

$$N_{abs}(t) = (P/E) \times F \times t$$

where,

$N_{abs}(t)$ = number of photons absorbed in time t by ICG

P = Optical power of the lamp

E = Energy required to activate the ICG in nm

t = irradiation time

$F = 1 - e^{-\alpha\rho l}$ (absorption factor)

here, α, ρ, and l are molar attenuation coefficient, density, and path length of the ICG.

The number of ROS produced by the ICG in time t were calculated by multiplying the quantum yield of the ICG to the number of photons absorbed in time t calculated in the previous step.

$$N_{ROS}(t) = \eta \times N_{abs}(t)$$

where,

$N_{ROS}(t)$ = number of ROS produced in time t,
$N_{abs}(t)$ = number of photons absorbed in time t,
η = quantum yield of standard photosensitizer

The number of ROS produced for the ICG was plotted against the ROS levels of ICG and slope of the line was calculated, which acts as the conversion factor (CF) to calculate the number of ROS produced for Dye 847-ZnO NPs.

$$N_{ROS}(t)^* = CF \times (ROS\ level_{Light}(t) - ROS\ level_{No\ Light})$$

where,

$N_{ROS}(t)^*$ = number of ROS produced by Dye 847-ZnO NPs in time t,
ROS $level_{Light}(t)$ = level of ROS produced by Dye 847-ZnO NPs on NIR irradiation for time t using DCFDA,
ROS $level_{No\ Light}$ = level of ROS produced by Dye 847-ZnO NPs without NIR irradiation using DCFDA,

Finally, the quantum yield of the NPs was calculated by dividing the number of ROS produced by the NPs in time t by the number of photons emitted by the NIR lamp for time t.

Table S2. Calculated Quantum yield (η) of Dye 847, Dye 847-ZnO and PEG-Dye 847-ZnO NPs at different concentrations (20 and 100 μg/mL) after exposure to irradiation (2 min NIR lamp) by comparing with the quantum yield of a standard photosensitizer (Indocyanine Green (ICG, η = 0.40)).

Dye 847 20 μg/mL	Dye 847 100 μg/mL	Dye 847-ZnO 20 μg/mL	PEG Dye 847-ZnO 20 μg/mL	Dye 847-ZnO 100 μg/mL	PEG-Dye 847-ZnO 100 μg/mL
**0.35**	0.50	0.40	0.32	0.68	0.60

© 2019 by the authors. Submitted for possible open access publication under the terms and conditions of the Creative Commons Attribution (CC BY) license (http://creativecommons.org/licenses/by/4.0/).
